# Celiac Disease, Inflammation and Oxidative Damage: A Nutrigenetic Approach

**DOI:** 10.3390/nu4040243

**Published:** 2012-03-27

**Authors:** Gianna Ferretti, Tiziana Bacchetti, Simona Masciangelo, Letizia Saturni

**Affiliations:** 1 Department of Odontostomatologic and Specialistic Clinics Sciences, Polytechnic University of Marche, via Ranieri 65, 60100 Ancona, Italy; Email: giannaferretti@gmail.com; 2 Department of Life and Environmental Sciences, Polytechnic University of Marche, via Ranieri 65, 60100 Ancona, Italy; Email: t.bacchetti@univpm.it (T.B.); s.masciangelo@gmail.com (S.M.); 3 Ibero-American University Foundation—FUNIBER, via Ranieri 65, 60100 Ancona, Italy

**Keywords:** celiac disease, gliadin peptides, nutrigenetic, nutrigenomic, proteomic, fitonutrients, oxidative stress, inflammation

## Abstract

Celiac disease (CD), a common heritable chronic inflammatory condition of the small intestine caused by permanent intolerance to gluten/gliadin (prolamin), is characterized by a complex interplay between genetic and environmental factors. Developments in proteomics have provided an important contribution to the understanding of the biochemical and immunological aspects of the disease and the mechanisms involved in toxicity of prolamins. It has been demonstrated that some gliadin peptides resistant to complete proteolytic digestion may directly affect intestinal cell structure and functions by modulating gene expression and oxidative stress. In recent years, the creation of the two research fields Nutrigenomics and Nutrigenetics, has enabled the elucidation of some interactions between diet, nutrients and genes. Various dietary components including long chain ω-3 fatty acids, plant flavonoids, and carotenoids have been demonstrated to modulate oxidative stress, gene expression and production of inflammatory mediators. Therefore their adoption could preserve intestinal barrier integrity, play a protective role against toxicity of gliadin peptides and have a role in nutritional therapy of celiac disease.

## Abbreviations

AA: arachidonic acid
COX-2: cycloxygenase-2
cPLA2: cytosolic phospholipase A2
CD: celiac disease
DHA: docosahexaenoic acid
EPA: eicosapentaenoic acid
GDF: gluten free diet
EGFR: epidermal growth factor receptor
HLA: human leukocytes antigen
IFN-γ: interferon-gamma
GSSG: oxidised glutathione
GSH: reduced glutathione
NO: nitric oxide
iNOS: inducible-nitric oxide synthase
PUFAs: polyunsaturated fatty acids
ROS: reactive oxygen species
RNS: reactive nitrogen species
TJ: tight junctions
tTG: tissue transglutaminase

## 1. Introduction

Celiac disease (CD), a common heritable chronic inflammatory condition of the small intestine caused by permanent intolerance to gluten/gliadin (prolamin), is characterized by a complex interplay between genetic and environmental factors [1,2]. The prolamin fractions in cereal grains (gliadin in wheat and similar alcohol-soluble proteins in other cereals, secalin in rye, hordein in barley) are the environmental stimuli responsible for the development of intestinal damage associated with CD [2,3]. The classical presentation of CD as a pediatric predominant disorder, is characterized by small-intestinal villous atrophy and crypt hyperplasia [4,5]. However especially in adult-onset patients, a preserved mucosal architecture characterized by dense lymphocytic infiltration, with no villous atrophy or crypt hyperplasia can also be observed.

An interplay between innate and adaptive immune responses to ingested gluten [5,6] is involved in CD. Developments in proteomics have provided an important contribution to the understanding of the biochemical and immunological aspects and the mechanisms involved in the toxicity of prolamins [7]. It has been demonstrated that the gliadin sequence contains regions which play a special role in CD pathogenesis: exert a cytotoxic activity or immunomodulatory activity. Other regions trigger oxidative stress and induce the release of pro-inflammatory cytokines [8,9]. As a consequence, elimination of gluten from the diet (gluten free diet, GDF) results in a clinical improvement in CD patients. 

A previous celiac disease genome-wide association study (GWAS) demonstrated risk variants in the human leukocytes antigen (HLA) region [10,11]. HLA-DQ2 and HLA-DQ8 risk alleles are necessary, but not sufficient, for celiac disease development. Recent studies have shown that 6% of the European and USA population do not present these alleles [12,13]. Therefore other no-HLA risk genes need to be investigated. 

Several dietary components exert anti-inflammatory and antioxidant roles and have a protective effect on intestinal epithelium [14], therefore their adoption could contribute to preserving intestinal barrier integrity and play a protective role against the toxicity of gliadin peptides in CD subjects [15]. In recent years, the creation of the two research fields “Nutrigenomics” and “Nutrigenetics”, has enabled the elucidation of some interactions between diet, nutrients and genes. “Nutrigenetics” refers to genetically determined differences in reactivity of individuals to specific foods, while “Nutrigenomics” refers to the functional interactions of food with the genome. The aim of the review is to summarize the approach of “Omic” sciences to elucidate the complex relationships between dietary factors, genetic polymorphisms and the gut structure as well as the functions in celiac disease.

## 2. Molecular Mechanisms on the Toxic Effects of Gluten

Usually food proteins are degraded into small peptides and aminoacids by peptidases before they can be transported across the epithelium. The high proline (Pro or P) content in gliadin and similar proteins of wheat and related cereals [6], renders these proteins resistant to complete proteolytic digestion in the human intestine. Therefore toxic oligopeptides with high concentration of proline and glutamine (Gln or Q) are accumulated in the small intestine and can exert toxic effects in genetically susceptible subjects [16]. 

**Figure 1:**
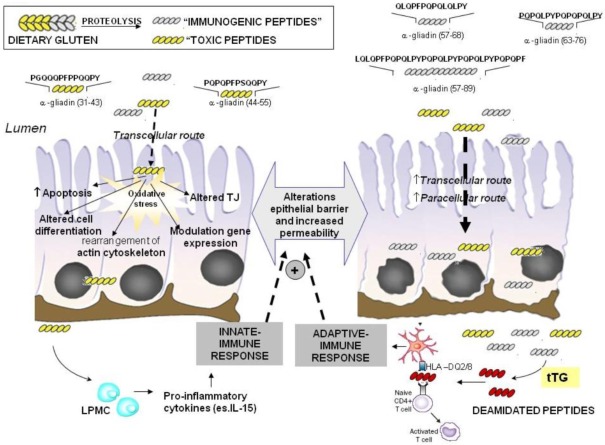
Intestinal epithelial damage in celiac disease: role of “toxic” and “immunogenic” peptides. “Toxic” peptides in intestinal cells induce tight junction (TJ) dysfunctions and several cytotoxic effects such as apoptosis and altered cell differentiation. Most of these effects are mediated by increased oxidative stress induced by gluten peptides in enterocytes. These alterations reflect in an impairment of the epithelial barrier and increased permeability. As a consequence, both “toxic” and “immunogenic” peptides of gliadin pass through the enterocytes leading to activation of the immune response (native and adaptive) contributing to cell damage and villous atrophy in celiac disease (CD) subjects. LPMC, lamina propria mononuclear cells; tTG, Tissue transglutaminase.

Different gluten peptides are involved in the CD process [8,9]. Computer modeling studies have demonstrated that two groups of biologically-active peptides derive from α-gliadin. The serine-containing group of peptides appears to be essentially cytotoxic, whilst the tyrosine-containing group has the capacity to trigger immunological reactions in CD patients [17]. Both types of activity in celiac disease are possible if there is defective digestion of the active peptides. As far as concerns the serine-containing peptides (Ser or S), their activity is linked to the presence of PSQQ and QQQP motifs. The tyrosine-containing peptides (Tyr or Y), sequences such as QQPY and/or QPYP are associated with immunological activity and hence toxicity [7]. Some examples of peptides of α-gliadin that have been widely investigated are P31–43, P31–49, P44–55, P57–68, P57–89, and P63–76 (Figure 1) [8]. 

### 2.1. Imunomodulatory Effects of Gluten Peptides

The “immunogenic” peptides such as P57–68, P57–89, and P63–76 cause an adaptive response that proceeds through their binding to HLA-DQ2 or -DQ8 of antigen presenting cells and the subsequent stimulation of T-cells [18,19] (Figure 1). The repetitive presence of these residues makes them a preferred substrate of the enzyme tissue transglutaminase (tTG), whose main function is to catalyse the covalent and irreversible cross-linking of a glutamine residue in glutamine-donor proteins with a lysine residue in glutamine-acceptor proteins, which results in the formation of DQ-“gluten” peptide complexes [20]. However, apart from cross-linking its substrates, tTG can also hydrolyse peptide-bound glutamine to glutamic acid either at a lower pH or when no acceptor proteins are available, a process leading to an enhanced immunogenicity of gluten peptides [1,6] (Figure 1). The DQ-“gluten” peptide complexes activate DQ2 or DQ8 restricted T-helper cells that proliferate and produce mainly Th1-type cytokines, particularly interferon-gamma (IFN-γ). The secretion of Th1 cytokines activates the release of enzymes such as matrix metalloproteinases that can damage the intestinal mucosa, with a loss of villous structure [21]. Th1 cytokines increase epithelial permeability which in turn will increase the passage of gluten peptides and peptide binding to DQ2 and DQ8 molecules on antigen-presenting cells, leading to a chronic feedback of the inﬂammatory process as long as gluten peptides are present in the intestinal lumen (Figure 1). The interactions between toxic gluten peptides and specific cells in lamina propria such as epithelial cells, macrophages and dendritic cells induce an innate immune response by up-regulating the expression of different mediators such as interleukin 15 (IL-15) with subsequent massive increase of intraepithelial lymphocytes [22]. These events contribute to the damage of the mucosal matrix [23] (Figure 1). In particular IL-15, a major mediator of the innate immune response is involved in proliferation of crypt enterocytes, an early alteration of CD mucosa causing crypts hyperplasia. 

### 2.2. No-Immune Mediated Cytotoxicity of Gluten: Effect on Oxidative Stress and Gene Expression

**Figure 2:**
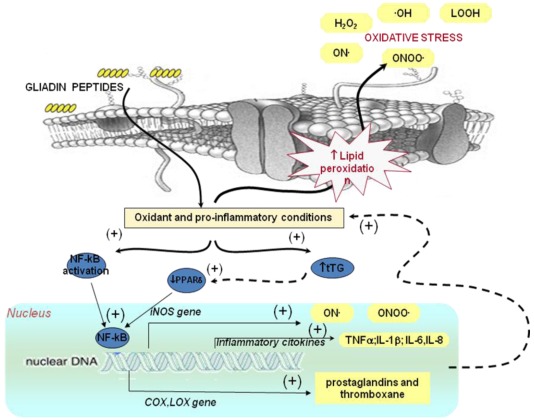
Effect of gluten on oxidative stress and gene expression. Alteration in oxidative balance induced by gliadin peptides in enterocytes is involved in the activation of transcription factor NF-κB. NF-κB activation induces the transcription of pro-inflammatory cytokines and enzymes such as COX2 and iNOS with consequent higher production of prostaglandins and NO metabolites contributing to the oxidative stress. The increased oxidative stress is also involved in the down-regulation of PPARγ mediated by tissue transglutaminase (tTG). The down-regulation of PPARγ may contribute to NF-κB activation. NF-κB, nuclear factor kappa-light-chain-enhancer of activated B cells; COX, cyclooxygenase; LOX, lipoxygenase; iNOS, inducible nitric-oxide synthase; PPARδ, peroxisome proliferator-activated receptors gamma; ONOO•, peroxynitrite; LOOH, lipid hydroperoxides; •OH, hydroxyl radical; H_2_O_2_ hydrogen peroxide.

In addition to “immunogenic” effects, gliadin peptides may directly affect intestinal cell structure and functions as demonstrated *in vitro* on cultured cells and intestine biopsies [24,25]. The effects exerted by “toxic peptides” P31–43, P31–49, P44–55 have been mainly investigated and different molecular mechanisms, strictly related, appear to be involved [24] (Figure 1 and Figure 2). 

#### 2.2.1. Effect of Gluten on Oxidative Stress

Some α-gliadin peptides, in particular P31–43 possess the ability to penetrate cells [25,26,27]. Thereby they are internalised by endocytic uptake [27]. Peptide P31–43 accumulation in lysosomes leads to activation of some signal transduction pathways and to increased levels of free radicals (reactive oxygen species, ROS and reactive nitrogen species, RNS) [28] (Figure 1 and Figure 2). Thereby it has been assumed that oxidative stress is one of the mechanisms that can play a role in gliadin toxicity. Using different cell models, it has been reported that gliadin exposure reflects in an intracellular oxidative imbalance, characterized by an increase in the levels of lipid peroxidation products (4-hydroxy-2(*E*)-nonenal (4-HNE)), an increase in the oxidised (GSSG)/reduced (GSH) glutathione ratio and a decrease of protein-bound sulfhydryl groups [29]. Significant structural perturbations of the cell plasma membrane were also detected [24,30]. The increase in oxidative damage could induce alterations of cell morphology [24], cell proliferation, apoptosis [31] and cell viability [32,33]. (Figure 1).

The relationship between oxidative damage and celiac diseases is supported by several studies on intestinal cells and circulating cells (Table 1). Increased levels of prostaglandin E2 (PGE_2_) were demonstrated in homogenized small bowel biopsy specimens from patients with active celiac disease [34] while the levels of the antioxidant enzyme glutathione peroxidase and reductase were decreased in biopsies from celiac disease patients with consequent decreased levels of GSH [35,36,37] (Table 1). Several investigations reported that reactive nitrogen species such as nitric oxide (NO) also play an important role in the pathogenesis of celiac disease with enhanced NO. Inducible-nitric oxide synthase (iNOS) is constitutively expressed in human duodenal enterocytes; its activity is increased in patients with untreated celiac disease [38,39,40] and is partially corrected when patients are treated with GFD [41,42]. High levels of NO are present in serum [43,44,45,46,47] and urine [48,49,50] of children with celiac disease and correlate with an increased expression of iNOS in the small intestine (Table 1). 

**Table 1:** Alterations in the levels of markers of oxidative stress and of antioxidant enzymes in intestine biopsies, blood and urine from celiac subjects (↑ increase; ↓ decrease).

**Intestine (intestinal biopsies)**	↑ level of lipid hydroperoxides (LOOH)
	↓ reduced glutathione (GSH) levels
	↓ glutathione peroxidase (GPx) activity
	↓ glutathione reductase (GR) activity
	↑ superoxide dismutase (SOD) activity
	↓ paraoxonase-1 (PON1) and paraoxonase-3 (PON3) expression
	↑ inducible-nitric oxide synthase (iNOS) expression
**Blood (plasma and blood cells)**	↑ lipid hydroperoxides (LOOH) level in plasma
	↑ thiobarbituric acid-reactive substances levels in plasma and lipoproteins
	↑ carbonyl groups levels in plasma
	↑ 8-hydroxyguanosine (8-oxodG) in DNA in leukocytes
	↑ NO metabolite levels plasma
	↑ nitrotyrosine levels in plasma
	↓ reduced glutathione levels in plasma
	↑ blood superoxide dismutase (SOD) activity
	↓ blood glutathione peroxidase (GPx) activity
	↓ blood glutathione reductase (GR) activity
	↓ alpha-tocopherol levels in plasma and in erythrocytes
	↓ plasma ascorbic acid levels
	↓ plasma retinol levels
**Urine**	↑ 8-hydroxyguanosine (8-oxodG) in DNA metabolite levels

An increase of markers of oxidative damage of lipids (thiobarbituric acid-reactive substances and lipid hydroperoxides), proteins (carbonyl groups) and DNA was demonstrated in intestinal cells and biological fluids [51,52,53,54] of CD with respect to controls (Table 1). Changes in vitamin E and antioxidant enzyme activities were observed also in circulating cells and plasma of patients [49,50,51,52,53,54,55] (Table 1). A decreased expression of the antioxidant and anti-inflammatory enzymes PON1 and PON3 mRNA was reported by Rothem *et al.* in intestinal biopsy of celiac patients [56]. The decreased antioxidant defenses may compromise the inflamed mucosa, rendering it more susceptible to oxidative tissue damage, hindering recovery of the mucosa and return of epithelial cell layer integrity. 

#### 2.2.2. Effect of Gluten on Gene Expression: Relationship with Oxidative Stress and Inflammation

Several studies have demonstrated that gliadin peptides are able to modulate gene expression in several cellular models [57,58]. The increased levels of ROS is involved in the reduced degradation of tTG by the ubiquitine-proteasome system, thus leading to increased tTG protein levels in susceptible targets as coeliac mucosa. An uncontrolled activation of the ROS-tTG axis induced by P31–43 leads to down-regulation of PPARγ with a derangement of the appropriate control of inflammation [59,60] (Figure 2). A down-regulation of PPARγ in CD subjects is confirmed by proteomic analysis of duodenal biopsies from celiac subjects [61]. In fact PPARγ receptor produced by several cell types, including epithelial cells, negatively regulates inflammatory gene expression by “transrepressing” inflammatory responses and even by modulating oxidative stress [28,59]. The increased secretion of inflammatory cytokines may, in turn, derange intestinal permeability and enhance the toxic effects of environmental triggers [57]. It has been hypothesized that the down-regulation of PPARγ may also contribute to NF-κB activation (Figure 2). In celiac disease, pro-inflammatory cytokines, adhesion molecules, and enzymes whose gene expression is known to be regulated by NF-κB are involved [57,62]. The synthesis of NF-κB and other transcription factors is dependent also on the intracellular redox state [63] and can promote synergistically transcriptional activity of pro-inflammatory genes [64]. In fact an increased expression of cycloxygenase-2 (COX-2), cytosolic phospholipase A2 (cPLA2) activity and IL-8 release in culture medium have been observed in cells incubated in the presence of gliadin peptides [58,65]. Moreover, in IFN-γ-stimulated RAW 264.7 macrophages, gliadin increases iNOS gene expression through a mechanism involving NF-κB and other transcription factors (IRF-1 and STAT-1α) [57,66] (Figure 2). An increased expression of several cytokines was observed in the epithelium of patients with active celiac disease [67,68]. Moreover, as aforementioned, patients with active CD and those on a gluten free diet have significantly higher levels of proinflammatory cytokines in the plasma, such as INF-γ, interleukin (IL)-1β, tumor necrosis factor-α (TNF-α), IL-6 and IL-8 compared to no celiac subjects [69].

The association between gluten, oxidative stress, gene expression and inflammation is supported by recent studies. The analysis of gene expression in small-bowel mucosal biopsy samples from untreated celiac disease patients using cDNA microarray has shown that about nine of 30 genes involved in cell signaling and differentiation were modified (receptor tyrosine kinase pathway starting from the epithelial growth factor receptor) with respect to control samples [70]. Using a three-dimensional epithelial cell differentiation model, it has been demonstrated that expression of about 29 genes is modified following the reaction to gluten. It has to be stressed that nine affected genes are involved directly or indirectly in the intracellular epidermal growth factor receptor (EGFR) signaling pathway. Therefore it has been hypothesized that a peptic tryptic digest of gluten and small gluten peptides elicits EGF-like responses on epithelial cells [71]. Removal of gluten from the ingested diet resulted in reversion in the transcription of 29 of 30 genes in the small-bowel biopsy samples. 

## 3. Nutritional Genomics in Celiac Disease

**Figure 3:**
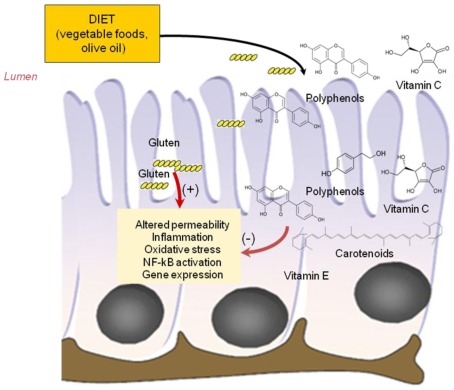
Protective effect of phytonutrients on cytotoxicity of gluten peptides. Antioxidant vitamins (vitamin C and E), polyphenols and carotenoids from dietary vegetable food and oil, were absorbed by intestinal cells and could exert protective effects against toxic effects exerted by gluten peptides on intestinal cells.

Various dietary components including plant polyphenols, carotenoids and fatty acids, have the potential to modulate predisposition to intestinal chronic inflammatory conditions and may have a role in nutritional therapy of celiac disease [14,72]. These components act through a variety of mechanisms including decreasing inflammatory mediator production through effects on cell signalling and gene expression, reducing the production of damaging oxidants and promoting gut barrier function and anti-inflammatory responses (Figure 3). 

### 3.1. Antioxidant Vitamins

Vitamin C and E are able to modulate immune responses in several ways, for instance by modulating leukocyte function and lymphocyte proliferation [73]. Vitamin C and E exert also antioxidant activity, and thereby modulate the inflammatory process. A decreased NF-κB activation with a consequent decreased release of pro-inflammatory cytokines (IL-8 and PAI-1) in the presence of vitamin E (particularly γ-tocopherol) has also been shown [74].

Bernardo *et al.* (2011) have recently reported that administration of vitamin C in small-bowel mucosal biopsy organ culture system prevents the augmented secretion IFN-γ, TNF-α, and IL-6 and increases the expression of IL-15 triggered by gliadin, suggesting that vitamin C supplementation might be beneficial for celiac patients [75].

### 3.2. Phyochemicals: Polyphenols and Carotenoids

Fruit and vegetables contain several polyphenols and carotenoids that produce distinct biological effects on the intestinal epithelial cells. Polyphenols and carotenoids exert antioxidant and anti-inflammatory properties. A likely target for these compounds seems to be the signal transduction cascade leading to the activation of transcription factors such as NF-κB [76,77,78]. Flavonoids have anti-inflammatory activity through several action mechanisms involving the reduction in the concentration of prostanoids and leukotrienes through the inhibition of eicosanoid generating enzymes such as phospholipase A2, COX and LOX and the inhibition of iNOS induction and expression in different cell models [14,78]. Also carotenoids are able to inhibit the expression of inflammation-related enzymes/proteins partly by suppressing activation of NF-κB, an effect potentially mediated by the inhibition of different protein kinases (e.g., mitogen-activated protein kinase; extracellular signal-regulated kinase) involved in signal transduction pathway [13,77]. 

Other authors have reported that lycopene, quercetin, and tyrosol decrease iNOS and COX-2 gene expression induced by gliadin in RAW 264.7 macrophages stimulated with IFN-γ [79]. The inhibition of iNOS and COX-2 gene expression occurred at transcriptional level by preventing NF-κB, IRF-1 and STAT-1α activation and was correlated with the inhibition of reactive oxygen species generation induced by gliadin and IFN-γ [79].

Flavonoids such as epigallocatechin gallate, genistein, myricetin and quercetin have also a protective effect on intestinal TJ barrier function [80]. As aforementioned, the TJs have crucial roles in paracellular transport of gluten as well as in barrier function in the intestines. As reviewed by Suzuki *et al.* (2011), flavonoids ameliorate intestinal TJ barrier dysfunction induced by oxidative stress and by inflammatory cytokines [80]. Quercetin has been reported to enhance intestinal TJ barrier function through the assembly and expression of TJ proteins. The change in phosphorylation status is responsible for the quercetin-mediated assembly of TJ proteins [80]. 

### 3.3. Fatty Acids

Fatty acids can influence inflammation through a variety of mechanisms, including acting via cell surface and intracellular receptors/sensors that control inflammatory cell signaling and gene expression patterns [72]. Eicosanoids produced from *n*-6 fatty acids such as arachidonic acid (AA) have a pro-inflammatory role. Conversely, *n*-3 fatty acids such as eicosapentaenoic acid (EPA) gives rise to eicosanoids with anti-inflammatory properties [72]. An excellent example of nutrigenomics is the influence of *n*-3 fatty acids on gene expression. In particular *n*-3 fatty acids have been reported to inhibit the activation of the transcription factor NF-κB with consequent inhibition of pro-inflammatory cytokine production.; in contrast, saturated fatty acids, especially lauric acid (12:0), enhanced NF-κB activation in macrophages and dendritic cells [81].

The effect of fatty acids on gene expression could be mediated also via fatty acid sensors or receptors such as PPARγ. In fact polyunsaturated fatty acids (PUFAs) and their derivatives are endogenous ligands for PPAR-γ and it has been demonstrated that PUFA-induced PPAR-γ is associated with a reduction in production of pro-inflammatory cytokines (TNF-α and IL-6) [82].

As far as concerns celiac disease it has been demonstrated that the cytosolic phospholipase A2 (cPLA2)—dependent release of AA from the intra-epithelial lymphocytes after incubation with gliadin, contribute to lymphocytes cytolysis and to the immune response of celiac disease. Moreover, using a human intestinal epithelial cell line (Caco-2) exposed to gliadin peptides, it has been demonstrated that docosahexaenoic acid (DHA), a long chain *n*-3 PUFA, is able to counteract many of the proinflammatory effects of AA. In fact DHA prevented the AA release, cycloxygenase-2 expression, cPLA2 activity and prostaglandin E2 and interleukin-8 release in culture medium suggesting that DHA inhibits the AA release by these cells [83]. 

## 4. Conclusions

Celiac disease is characterized by a complex interaction between genetic and environmental factors. The mucosal damage in celiac patients is considered to be induced by an interplay between innate and adaptive immune responses to ingested gluten. Developments in proteomics have provided an important contribution to the understanding of the biochemical and immunological aspects and the mechanisms involved in toxicity of prolamins. Inflammation and oxidative stress due to an increase of reactive oxygen species and a decrease of antioxidant defenses are involved in the molecular mechanisms of celiac disease. This in turn leads to uncontrolled activation of the redox-sensitive, pro-inflammatory transcription factors NF-κB, continued production of ROS and RNS and support of chronic inflammation. 

Previous studies have demonstrated that several nutrients exert antioxidant effects and influence gene expression, therefore they represent a useful approach for nutritional intervention in CD subjects, as corroborated by recent *in vitro* studies that have demonstrated that phytonutrients (lycopene, quercitine, vitamin C and tyrosol) protect against the cytotoxic effect of gliadin. A protective effect has also been exerted by DHA. 

To realize the usefulness of nutritional genomics as a tool for targeted medical nutrition therapy, further basic research, extensive epidemiological studies and controlled intervention trials are needed to investigate whether long chain unsaturated fatty acids , antioxidant vitamins , plant polyphenols and carotenoids modulate *in vivo *predisposition of chronic inflammatory conditions and thus have a role in the therapy of celiac disease. 

## References

[1] Sollid L.M., Jabri B. (2005). Is celiac disease an autoimmune disorder?. Curr. Opin. Immunol..

[2] Van Heel D.A., West J. (2006). Recent advances in coeliac disease. Gut.

[3] Wieser H., Koehler P. (2008). The biochemical basis of celiac disease. Cereal Chem..

[4] Diosdado B., van Oort E., Wijmenga C. (2005). “Coelionomics”: Towards understanding the molecular pathology of coeliac disease. Clin. Chem. Lab. Med..

[5] Koning F., Schuppan D., Cerf-Bensussan N., Sollid L.M. (2005). Pathomechanisms in celiac disease. Best Pract. Res. Clin. Gastroenterol..

[6] Jabri B., Kasarda D.D., Green P.H.R. (2005). Innate and adaptive immunity: The Yin and Yang of celiac disease. Immunol. Rev..

[7] Mamone G., Picarello G., Addeo F., Ferranti P. (2011). Proteomic analysis in allergy and intolerance to wheat products. Expert Rev. Proteomics.

[8] Ciccocioppo R., di Sabatino A., Corazza G.R. (2005). The immune recognition of gluten in coeliac disease. Clin. Exp. Immunol..

[9] Shan L., Molberg Ø., Parrot I., Hausch F., Filiz F., Gray G.M., Sollid L.M., Khosla C. (2002). Structural basis for gluten intolerance in celiac sprue. Science.

[10] Trynka G., Zhernakova A., Romanos J., Franke L., Hunt K.A., Turner G., Bruinenberg M., Heap G.A., Platteel M., Ryan A.W. (2009). Coeliac disease-associated risk variants in TNFAIP3 and REL implicate altered NF-kappaB signalling. Gut.

[11] Vader W., Stepniak D., Kooy Y., Mearin L., Thompson A., van Rood J.J., Spaenij L., Koning F. (2003). The HLA-DQ2 gene dose effect in celiac disease is directly related to the magnitude and breadth of gluten-specific T cell responses. Proc. Natl. Acad. Sci. USA.

[12] Harmon G.S., Lebeck L.K., Weidner N. (2011). Gluten-dependent enteropathy and atypical human leukocyte antigen alleles. Hum. Pathol..

[13] Karell K., Louka A.S., Moodie S.J., Ascher H., Clot F., Greco L., Ciclitira P.J., Sollid L.M., Partanen J. (2003). European genetics cluster on celiac disease. Hum. Immunol..

[14] Calder P.C., Albers R., Antoine J.M., Blum S., Bourdet-Sicard R., Ferns G.A., Folkerts G., Friedmann P.S., Frost G.S., Guarner F. (2009). Inflammatory disease processes and interactions with nutrition. Br. J. Nutr..

[15] Matysiak-Budnik T., Candalh C., Dugave C., Namane A., Cellier C., Cerf-Bensussan N., Heyman M. (2003). Alterations of the intestinal tran sport and processing of gliadin peptides in celiac disease. Gastroenterology.

[16] Trynka G., Wijmenga C., van Heel D.A. (2010). A genetic perspective on coeliac disease. Trends Mol. Med..

[17] Cornell H.J., Wills-Johnson G. (2001). Structure-activity relationships in coeliac-toxic gliadin peptides. Amino Acid.

[18] Auricchio S., Barone M.V., Troncone R. (2004). Dietary proteins and mechanismsof gastrointestinal diseases: Gliadin as a model. J. Pediatr. Gastroenterol. Nutr..

[19] Hausch F., Shan L., Santiago N.A., Gray G.M., Khosla C. (2002). Intestinal digestive resistance of immunodominant gliadin peptides. Am. J. Physiol. Gastrointest. Liver Physiol..

[20] Folk J.E. (1983). Mechanism and basis for specificity of transglutaminasecatalyzede-(g-glutamyl) lysine bond formation. Adv. Enzymol. Relat. Areas Mol. Biol..

[21] Kagnoff M.F. (2005). Overview and pathogenesis of celiac disease. Gastroenterology.

[22] Maiuri L., Ciacci C., Ricciardelli I., Vacca L., Raia V., Auricchio S., Picard J., Osman M., Quarantino S., Londei M. (2003). Association between innate response to gliadin and activation of pathogenic T cells in celiac disease. Lancet.

[23] Pender S.L.F., Lionetti P., Murch S.H., Wathan N., MacDonald T.T. (1996). Proteolytic degradation of intestinal mucosa extracellular matrix after lamina propria T cell activation. Gut.

[24] Elli L., Dolfini E., Bardella M.T. (2003). Gliadin cytotoxicity and *in vitro* cell cultures. Toxicol. Lett..

[25] Maiuri L., Troncone R., Mayer M., Coletta S., Picarelli A., de Vincenzi M., Pavone V., Auricchio S. (1996). *In vitro* activities of A-gliadin related synthetic peptides: Damaging affect on the atrophic coeliac mucosa and activation of mucosal immune response in the treated coeliac mucosa. Scand. J. Gastroenterol..

[26] Schumann M., Richter J.F., Wedell I., Moos V., Zimmermann-Kordmann M., Schneider T., Daum S., Zeitz M., Fromm M., Schulzke J.D. (2008). Mechanisms of epithelial translocation of the a2-gliadin-33mer in coeliac sprue. Gut.

[27] Heyman M., Menard S. (2009). Pathways of gliadin transport in celiac disease. Ann. N. Y. Acad. Sci..

[28] Zimmer K.P., Fischer I., Mothes T., Weissen-Plenz G., Schmitz M., Wieser H., Büning J., Lerch M.M., Ciclitira P.C., Weber P. (2010). Endocytotic segregation of gliadin peptide 31–49 in enterocytes. Gut.

[29] Luciani A., Villella V.R., Vasaturo A., Giardino I., Pettoello-Mantovani M., Guido S., Cexus O.N., Peake N., Londei M., Quaratino S. (2010). Lysosomal accumulation of gliadin p31–43 peptide induces oxidative stress and tissue transglutaminase-mediated PPARgamma downregulation in intestinal epithelial cells and coeliac mucosa. Gut.

[30] Rivabene R., Mancini E., de Vincenzi M. (1453). *In vitro* cytotoxic effect of wheat gliadin-derived peptides on the Caco-2 intestinal cell line is associated with intracellular oxidative imbalance: Implications for coeliac disease. Biochim. Biophys. Acta.

[31] Di Sabatino A., Ciccocioppo R., D’Alò S., Parroni R., Millimaggi M., Cifone M.G., Corazza G.R. (2001). Intraepithelial and lamina propria lymphocytes show distinct patterns of apoptosis whereas both populations are active in Fas based cytotoxicity in coeliac disease. Gut.

[32] Giovannini C., Sanchez M., Straface E., Scazzocchio B., Silano M., de Vincenzi M. (2000). Induction of apoptosis in CaCo-2 cells by wheat gliadin peptides. Toxicology.

[33] Giovannini C., Mancini E., de Vincenzi M. (1996). Inhibition of the cellular metabolism of Caco-2 cells by prolamin peptides from cereals toxic for coeliacs. Toxicol. In Vitro.

[34] Giovannini C., Maiuri L., de Vincenzi M. (1994). Mytotoxic effect of prolamin-derived peptides on *in vitro* cultures of cell line Caco-2: Implications for coeliac disease. Toxicol. In Vitro.

[35] Dolfini E., Elli L., Dasdia T., Bufardeci B., Colleoni M.P., Costa B., Floriani I., Falini M.L., Guerrieri N., Forlani F. (2002). *In vitro* cytotoxic effect of bread wheat gliadin on the LoVo human adenocarcinoma cell line. Toxicol. In Vitro.

[36] Lavö B., Knutson L., Lööf L., Hällgren R. (1990). Gliadin challenge-induced jejunal prostaglandin E2 secretion in celiac disease. Gastroenterologist.

[37] Stojiljković V., Todorović A., Pejić S., Kasapović J., Saicić Z.S., Radlović N., Pajović S.B. (2009). Antioxidant status and lipid peroxidation in small intestinal mucosa of children with celiac disease. Clin. Biochem..

[38] Stahlberg M.R., Hietanen E., Maki M. (1988). Mucosal biotransformation rates in the small intestine of children. Gut.

[39] Sido B., Hack V., Hochlehnert A., Lipps H., Herfarth C., Droge W. (1998). Impairment of intestinal glutathione synthesis in patients with inflammatory bowel disease. Gut.

[40] Daniels I., Cavill D., Murray I.A., Long R.G. (2005). Elevated expression of iNOS mRNA and protein in coeliac disease. Clin. Chim. Acta.

[41] Ter Steege J., Buurman W., Arends J.W., Forget P. (1997). Presence of inducible nitric oxide synthase, nitrotyrosine, CD68, and CD14 in the small intestine in celiac disease. Lab. Invest..

[42] Beckett C.G., Dell’Olio D., Ellis H.J., Rosen-Bronson S., Ciclitira P.J. (1998). The detection and localization of inducible nitric oxide synthase production in the small intestine of patients with coeliac disease. Eur. J. Gastroenterol. Hepatol..

[43] Holmgren Peterson K., Falth-Magnusson K., Magnusson K.E., Stenhammar L., Sundqvist T. (1998). Children with celiac disease express inducible nitric oxide synthase in the small intestine during gluten challenge. Scand. J. Gastroenterol..

[44] Murray I.A., Daniels I., Coupland K, Smith J.A., Long R.G. (2002). Increased activity and expression of iNOS in human duodenal enterocytes from patients with celiac disease. Am. J. Physiol. Gastrointest. Liver Physiol..

[45] Murray I.A., Bullimore D.W., Long R.G. (2003). Fasting plasma nitric oxide products in coeliac disease. Eur. J. Gastroenterol. Hepatol..

[46] Ter Steege J.C., Koster-Kamphuis L., van Straaten E.A., Forget P.P., Buurman W.A. (1998). Nitrotyrosine in plasma of celiac disease patients as detected by a new sandwich ELISA. Free Radic. Biol. Med..

[47] Ertekin V., Selimoğlu M.A., Türkan Y., Akçay F. (2005). Serum nitric oxide levels in children with celiac disease. J. Clin. Gastroenterol..

[48] Van Straaten E.A., Koster-Kamphuis L., Bovee-Oudenhoven I.M., van der Meer R., Forget P.P. (1999). Increased urinary nitric oxide oxidation products in children with active coeliac disease. Acta Paediatr..

[49] Högberg L., Webb C., Fälth-Magnusson K., Forslund T., Magnusson K.E., Danielsson L., Ivarsson A., Sandström O., Sundqvist T. (2011). Children with screening-detected coeliac disease show increased levels of nitric oxide products in urine. Acta Paediatr..

[50] Sundqvist T., Laurin P., Fälth-Magnusson K., Magnusson K.E., Stenhammar L. (1998). Significantly increased levels of nitric oxide products in urine of children with celiac disease. J. Pediatr. Gastroenterol. Nutr..

[51] Stojiljković V., Todorović A., Radlović N., Pejić S., Mladenović M., Kasapović J., Pajović S.B. (2007). Antioxidant enzymes, glutathione and lipid peroxidation in peripheral blood of children affected by coeliac disease. Ann. Clin. Biochem..

[52] Odetti P., Valentini S., Aragno I., Garibaldi S., Pronzato M.A., Rolandi E., Barreca T. (1998). Oxidative stress in subjects affected by celiac disease. Free Radic. Res..

[53] Lavy A., Ben Amotz A., Aviram M. (1993). Increased susceptibility to undergo lipid peroxidation of chylomicrons and low-density lipoprotein in celiac disease. Ann. Nutr. Metab..

[54] Szaflarska-Poplawska A., Siomek A., Czerwionka-Szaflarska M., Gackowski D., Rózalski R., Guz J., Szpila A., Zarakowska E., Olinski R. (2010). Oxidatively damaged DNA/oxidative stress in children with celiac disease. Cancer Epidemiol. Biomark. Prev..

[55] Hozyasz K.K., Chelchowska M., Laskowska-Klita T. (2003). Vitamin E levels in patients with celiac disease. Med. Wieku Rozwoj..

[56] Rothem L., Hartman C., Dahan A., Lachter J., Eliakim R., Shamir R. (2007). Paraoxonases are associated with intestinal inflammatory diseases and intracellularly localized to the endoplasmic reticulum. Free Radic. Biol. Med..

[57] Maiuri M.C., de Stefano D., Mele G., Iovine B., Bevilacqua M.A., Greco L., Auricchio S., Carnuccio R. (2003). Gliadin increases iNOS gene expression in interferon—Stimulated RAW 264.7 cells through a mechanism involving NF-κB. Naunyn Schmiedebergs Arch. Pharmacol..

[58] de Stefano D., Maiuri M.C., Iovine B., Ialenti A., Bevilacqua M.A., Carnuccio R. (2006). The role of NF-κB, IRF-1, and STAT-1α transcription factors in the iNOS gene induction by gliadin and IFN-γin RAW 264.7 macrophages. J. Mol. Med..

[59] Thomas K.E., Sapone A., Fasano A., Vogel S.N. (2006). Gliadin stimulation of murine macrophage inflammatory gene expression and intestinal permeability are MyD88-dependent: Role of the innate immune response in celiac disease. J. Immunol..

[60] Maiuri L., Ciacci C., Ricciardelli I., Vacca L., Raia V., Rispo A., Griffin M., Issekutz T., Quaratino S., Londei M. (2005). Unexpected role of surface transglutaminase type II in celiac disease. Gastroenterology.

[61] Luciani A., Villella V.R., Vasaturo A., Giardino I., Pettoello-Mantovani M., Guido S., Cexus O.N., Peake N., Londei M., Quaratino S. (2010). Lysosomal accumulation of gliadin p31e43 peptide induces oxidative stress and tissue transglutaminase-mediated PPARg downregulation in intestinal epithelial cells and coeliac mucosa. Gut.

[62] de Re V., Simula M.P., Notarpietro A., Canzonieri V., Cannizzaro R., Toffoli G. (2010). Do gliadin and tissue transglutaminase mediate PPAR downregulation in intestinal cells of patients with coeliac disease?. Gut.

[63] Simula M.P., Cannizzaro R., Canzonieri V., Pavan A., Maiero S., Toffoli G., de Re V. (2010). PPAR signaling pathway and cancer-related proteins are involved in celiac disease-associated tissue damage. Mol. Med..

[64] Maiuri M.C., de Stefano D., Mele G., Fecarotta S., Greco L., Troncone R., Carnuccio R. (2003). Nuclear factor kappa B is activated in small intestinal mucosa of celiac patients. J. Mol. Med..

[65] Flohè L., Brigelius-Flohe R., Saliou C., Traber M.G., Packer L. (1997). Redox regulation of NF-κB activation. Free Radic. Biol. Med..

[66] Ohmori Y., Hamilton T.A. (1993). Cooperative interaction between interferon (IFN) stimulus response element and kappa B sequence motifs controls IFN gamma- and lipopolysaccharide-stimulated transcription from the murine IP 10 promoter. J. Biol. Chem..

[67] Vincentini O., Quaranta M.G., Viora M., Agostoni C., Silano M. (2011). Docosahexaenoic acid modulates *in vitro* the inflammation of celiac disease in intestinal epithelial cells via the inhibition of cPLA2. Clin. Nutr..

[68] Friis S., Anthonsen D., Norén O., Sjöström H. (1994). Gamma-type gliadins cause secretion of prostaglandin E2 in patients with coeliac disease. Clin. Chim. Acta.

[69] Juuti-Uusitalo K., Mäki M., Kaukinen K., Collin P., Visakorpi T., Vihinen M., Kainulainen H. (2004). cDNA microarray analysis of gene expression in coeliac disease jejunal biopsy samples. J. Autoimmun..

[70] Juuti-Uusitalo K., Mäki M., Kainulainen H., Isola J., Kaukinen K. (2007). Gluten affects epithelial differentiation-associated genes in small intestinal mucosa of coeliac patients. Clin. Exp. Immunol..

[71] Cataldo F., Lio D., Marino V., Scola L., Crivello A., Corazza G.R. (2003). Working Group of the SIGEP; Working Group of “Club del Tenue”. Plasma cytokine profiles in patients with celiac disease and selective IgA deficiency. Pediatr. Allergy Immunol..

[72] Calder P.C. (2011). Fatty acids and inflammation: The cutting edge between food and pharma. Eur. J. Pharmacol..

[73] Goetzl E.J., Wasserman S.I., Gigli I., Austen K.F. (1974). Enhancement of random migration and chemotactic response of human leukocytes by ascorbic acid. J. Clin. Invest..

[74] Cook-Mills J.M., McCary C.A. (2010). Isoforms of vitamin E differentially regulate inflammation. Endocr. Metab. Immune Disord. Drug Targets.

[75] Bernardo D., Martínez-Abad B., Vallejo-Diez S., Montalvillo E., Benito V., Anta B., Fernández-Salazar L., Blanco-Quirós A., Garrote J.A., Arranz E. (2012). Ascorbate-dependent decrease of the mucosal immune inflammatory response to gliadin in coeliac disease patients. Allergol. Immunopathol..

[76] Hecker M., Preiss C., Klemm V., Busse R. (1996). Inhibition by antioxidants of nitric oxide synthase expression in murine macrophages: Role of nuclear factor kappa B and interferon regulatory factor 1. Br. J. Pharmacol..

[77] Heber D., Lu Q.Y. (2002). Overview of mechanisms of action of lycopene. Exp. Biol. Med..

[78] Di Carlo G., Mascolo N., Izzo A.A., Capasso F. (1999). Flavonoids: Old and new aspects of a class of natural therapeutic drugs. Life Sci..

[79] de Stefano D., Maiuri M.C., Simeon V., Grassia G., Soscia A., Cinelli M.P., Carnuccio R. (2007). Lycopene, quercetin and tyrosol prevent macrophage activation induced by gliadin and IFN-gamma. Eur. J. Pharmacol..

[80] Suzuki T., Hara H. (2011). Role of flavonoids in intestinal tight junction regulation. J. Nutr. Biochem..

[81] Lee J.Y., Sohn K.H., Rhee S.H., Hwang D. (2001). Saturated fatty acids, but not unsaturated fatty acids, induce the expression of cyclooxygenase-2 mediated through Toll-like receptor 4. J. Biol. Chem..

[82] Zapata-Gonzalez F., Rueda F., Petriz J., Domingo P., Villarroya F., Diaz-Delfin J., de Madariaga M.A., Domingo J.C. (2008). Human dendritic cell activities are modulated by the omega-3 fatty acid, docosahexaenoic acid, mainly through PPAR(gamma):RXR heterodimers: Comparison with other polyunsaturated fatty acids. J. Leukoc. Biol..

[83] Vincentini O., Quaranta M.G., Viora M., Agostoni C., Silano M. (2011). Docosahexaenoic acid modulates *in vitro* the inflammation of celiac disease in intestinal epithelial cells via the inhibition of cPLA2. Clin. Nutr..

